# Towards Evidence-Based Food Safety Governance with Wastewater-Based Epidemiology (WBE) Technology in China

**DOI:** 10.3390/toxics12070504

**Published:** 2024-07-12

**Authors:** Xin Wei, Ying Xiong, Hongmei Huang, Xiqing Li, Lei Zhang

**Affiliations:** 1 Institute of Xnewera, Peking University, Beijing 100872, China; 2 School of Ecology & Environment, Renmin University of China, Beijing 100871, China; yingxiong@ruc.edu.cn

**Keywords:** food safety, pesticides residue governance, WBE, interdisciplinary officials, ecological and health risk assessment

## Abstract

Pesticide residues in food pose significant risks to public health and have long been a major concern in Chinese cities. The management of these risks is influenced by various factors, including the characteristics of responsible officials. This study tests the relationship between the levels of pesticide residues and the responsible officials’ interdisciplinary backgrounds and their tenure cycles, which is crucial for improving food safety governance in Chinese cities. Based on wastewater-based epidemiology (WBE) and data from 32 Chinese cities, it was found that the interdisciplinary backgrounds of officials had a significant negative relationship with urban pesticide residues in wastewater, indicating that the interdisciplinary knowledge background or working experience of officials in food safety-related agencies was associated with the supervision and control of urban pesticide residues. This study also generated evidence-based knowledge on how to improve food safety through assigning younger and interdisciplinary officials to the responsible governmental agencies, where WBE is more likely to be adopted.

## 1. Introduction

### 1.1. The Significance of Pesticide Residue Governance in Food Safety

The current literature on trust in food seems to be preoccupied with the paradox between increasingly stringent regulation on food safety and recurrent food safety accidents [1]. Although most experts claimed that food safety had been improved over the years through superior technologies, better monitoring devices, stricter control measures and more elaborate legal frameworks, these food safety arrangements seemed to have repeatedly failed to generate the necessary trust among consumers [2,3,4,5]. In 2012, food safety moved to the top of public concerns, next to environmental pollution [6]. A more recent survey reported that the food safety perception index remained low in cities across the country.

China’s 2009 Food Safety Law was the primary food regulation aimed to control national food quality and safety. The law consists of formal food safety standards, monitoring and control mechanisms, and various requirements for certified safe, green, and organic food. However, this regulation had been criticized for its lack of effectiveness [7,8]. Amendments to the 2009 Food Safety Law were made in 2015 and further in 2021 to make it one of the world’s most stringent of such laws. Nevertheless, a lack of timed, reliable, quantitative data has remained as a bottleneck for evidence-based food risk monitoring, risk assessment, and managerial performance evaluation.

Meanwhile, China has become the world’s largest producer and consumer of chemical fertilizers and pesticides. The total amount of pesticides used per year grew from 1.28 million tons in 2000 to 1.8 million tons in 2013, with an average annual increase of 2.7%. The intensity of pesticide use increased from 8 kg per ha in 2000 to 11 kg per ha in 2013, with an average annual growth of 2.3% [9]. The average utilization rates of fertilizers and pesticides are 33% and 35%, respectively, 15% to 30% lower than utilization rates in developed countries. To cope with the harms of the overuse of pesticides on the food production side, the Ministry of Agriculture implemented “Action to Achieve Zero Growth of Pesticide Use by 2020”, aiming to keep the use of pesticides per unit of land area below the average level in the years of 2012 to 2014, and striving to achieve zero growth in the total use of pesticides by 2020. Again, the effectiveness and effects of such action need to be evaluated based on reliable data to avoid a “data game”.

### 1.2. The Influence of Officials’ Characteristics on Pesticide Residue Governance

#### 1.2.1. The Impact of Responsible Officials’ Backgrounds on Policy Priority

The backgrounds of responsible officials, including their educational backgrounds and previous work experiences, can significantly influence their individual preferences, which, in turn, affect their priorities when dealing with various issues in their areas, such as economic development and other social public affairs (including environmental protection, food safety, etc.).

First, commercial experience can impact officials’ priorities when they enter politics. It is demonstrated that mayors with business experience are more likely to invest in infrastructure while reducing redistributive spending [10]. In Russia, it has been shown that politicians with a business background prioritize economic infrastructure over social infrastructure [11].

Second, apart from commercial backgrounds, most studies have focused on officials’ environmental backgrounds. Regarding educational background, it has been found that if a city-level “green official” has studied environmental science, environmental engineering, environmental economics, or related fields during their academic career, the city-level government’s environmental awareness and emphasis on green development can positively impact the emission reduction effects of grassroots government environmental constraints, ultimately reducing regional air pollution [12]. Concerning work experience, it has been shown that environmentally inclined politicians with prior environment-related work experience strategically leverage their expertise in environmental protection to allocate more effort to environmental causes, resulting in lower SO_2_ emission levels from firms located in their cities [13].

Third, only a limited amount of Chinese research has explored the impact of officials’ agricultural or food safety backgrounds on local development. It was discovered that the educated youth experience of governors and provincial party secretaries can increase local governments’ agricultural financial input, thus positively impacting rural revitalization [14]. It was found that officials with agriculture-related experience can significantly promote the development of geographical indication agricultural products, with the impact of agriculture-related educational experience being more significant than that of agriculture-related work experience, and gradually increasing over time [15]. In addition, the research on the officials’ food safety backgrounds is relatively scarce.

In conclusion, through reviewing the relevant literature, the knowledge gap is identified, which involves the impacts of the responsible officials’ agricultural or food safety backgrounds on their individual preference and policy priority. This highlights the necessity of conducting this research to investigate how officials’ agricultural or food safety expertise and experience influence their priorities and decision-making, ultimately affecting the pesticide residue levels in local food.

#### 1.2.2. The Impact of Responsible Officials’ Tenure Cycles on Policy Priority

The influence of political career cycles and incentives on the behavior of officials has been a popular research topic in China. As the most fundamental and influential work, it is established that the promotion prospects of officials are closely linked to their economic performance, creating strong incentives for them to prioritize growth-enhancing policies [16]. Building upon this, the crucial role of age in determining the career development of Chinese officials is highlighted, with older leaders more likely to face retirement or replacement. Subsequent research has delved deeper into the specific paths in which age intersects with career incentives to influence the priorities of Chinese officials [17].

First, officials under 55 have greater latitude to address a broader range of concerns beyond economic growth, as they have a relatively long political career ahead of them. it is noted that career advancement for these officials is often determined by their performance in lower-level positions, suggesting that they have a longer time horizon to balance economic development with other social issues [18]. Similarly, 55 is identified as a crucial age threshold, noting that officials not promoted beyond a certain level by this age face limited further advancement opportunities [19].

Second, officials between the ages of 55 and 57 face a unique set of pressures, as studies highlight that officials who fail to achieve a certain rank by the age of 55 are unlikely to be promoted to higher positions before reaching the mandatory retirement age. With limited time remaining in their careers, they may prioritize economic growth more heavily to enhance their prospects for advancement. This “sprinting” behavior, as described in the context of the age dilemma faced by officials over 55, likely applies particularly strongly to those in the 55–57 age range, who have a narrow window to achieve their career goals before facing mandatory retirement [20].

Third, officials over 57 and approaching the age of 60 may have weaker incentives to focus on either economic development or solving social problems. Since the mandatory retirement age in China is 60, officials may have less motivation to pursue ambitious policy goals in their remaining years in office.

The existing literature has primarily focused on the age dilemma surrounding the age of 55 years, with limited research investigating other different age brackets between 55 and 60. To fill this gap, this study follows the existing literature by categorizing officials into three distinct age groups: those younger than 55, those between 55 and 57, and those over 57 and approaching the mandatory retirement age of 60. By examining how these age and career prospects-based incentives translate into concrete differences in officials’ priorities, this study aims to capture the nuances of these dynamics in a more refined manner.

### 1.3. The Application of Wastewater-Based Epidemiology (WBE) as an Evidence-Based Approach

With the development of urban sewage infrastructure, urban wastewater has been increasingly recognized as an important source of information and reflection on society. Wastewater-based epidemiology (WBE), a new but fast-developing approach to estimate the level of a given substance, has demonstrated enormous potential to provide rapid, objective, and up-to-date information for urban risk governance. The WBE approach, firstly proposed by Daughton in 2001, involves measuring the concentrations of residues in wastewater and back-calculating consumption at local, national and/or international scales [21]. In recent years, the application of WBE has been expanded from monitoring the abuse of illicit drugs to evaluating the consumption of pharmaceuticals and exposure to plasticizers and pesticides [22,23,24]. A broad range of biomarkers in the wastewater can tell a lot about a particular population in almost real-time and with a high geographical resolution. Nevertheless, the majority of studies into biomarkers are still academic and exploratory in nature. It is believed that the analysis of sewage will be capable of delivering a wealth of socially relevant information.

The pesticide residue in food remains one of the top public concerns of urban consumers in China. Responsible city managers need to understand the pesticide residues in agricultural products consumed in cities and the risk of pesticide exposure for city residents. Monitoring the risk of pesticide residues in a city should be a fundamental component of the Smart City sense system and the foundation of Big Data for urban health. The demonstrated success of WBE in drug monitoring and management performance evaluation in a few Chinese cities inspired researchers to apply WBE for testing pesticides in urban wastewater and further explore how these data can serve as a “game changer” in current food safety governance in Chinese cities [24]. This study attempted, based on data from 32 Chinese cities, to shed light on the relationship between the levels of pesticide residues and the officials’ trans-sectoral working experience and tenure cycles, with an aim to develop strategies of promoting the application of WBE-based pesticide residue monitoring in cities to produce safety governance.

Since pesticides are highly water-soluble, the residues on vegetables and fruits that enter the urban “vegetable basket” will enter domestic sewage during the cleaning process, and then enter sewage treatment plants through sewage pipe networks. Most of the pesticides in urban domestic sewage come from the discharge of residents washing fruits and vegetables. Therefore, sewage samples can be collected at the inlet of sewage treatment plants, and the concentration of pesticides can be analyzed through advanced detection technologies. By combining this with parameter model calculations, the residues of these pesticides can be inferred. WBE is the most direct data source for measuring the actual level of pesticide residues and assessing the potential exposure risk to residents. It can not only analyze and evaluate the overall level of pesticide residues in cities, but also identify areas with prominent pesticide residues, which has important reference significance for food safety management departments and pesticide management departments.

To conclude, effective urban pesticide control is crucial for ensuring public health and environmental safety. This study aims to investigate how the interdisciplinary backgrounds and tenure cycles of urban government officials impact their prioritization in implementing pesticide-related regulations, with the application of WBE as an evidence-based approach.

**Hypothesis 1.**  *The interdisciplinary backgrounds of urban government officials significantly influences the effectiveness of urban pesticide control measures.*

**Hypothesis 2.**  *The stages of the tenure cycles of urban government officials significantly affect their prioritization of long-term social issues, such as food safety.*

## 2. Materials and Methods

### 2.1. Data Sampling 

#### 2.1.1. Wastewater Sampling

Wastewater samples were collected from 32 major Chinese cities distributed in all seven geographic regions of China: Harbin (HRB), Changchun (CC) and Shenyang (SY) of Northeast China; Beijing (BJ), Shijiazhuang (SJZ), Hohhot (HO) and Taiyuan (TY) of North China; Lanzhou (LZ), Yinchuan (YC), Xining (XN), Urumqi (UM) and Xi’an (XA) of Northwest China; Zhengzhou (ZZ) and Wuhan (WH) of Central China; Shanghai (SH), Nanjing (NJ), Suzhou (SUZ), Hefei (HF), Hangzhou (HZ), Nanchang (NC), Jinan (JN), Qingdao (QD), Fuzhou (FZ) and Xiamen (XM) of East China; Kunming (KM), Chengdu (CD), Guiyang (GY) and Chongqing (CQ) of Southwest China; Haikou (HAK), Shenzhen (SZ), Zhongshan (ZS) and Nanning (NN) of South China. Of these cities, twenty-seven are provincial capitals or municipalities directly under the central government (BJ, SH, and CQ). The other five cities (QD, SuZ, XM, ZS, and SZ) are equivalent to provincial capitals in terms of economic development and population sizes.

In total, 119 wastewater treatment plants (WWTPs) were selected for sampling in the above 32 cities, and two or more WWTPs were selected in most sampled cities. The WWTPs selected in this study are the major domestic sewage treatment plants in the cities, to ensure that the samples collected can fully represent the situation of the city. The sum of the population served by these WWTPs is 330 million, accounting for 23% of the entire population of the country.

The sampling campaign was conducted from August 2017 to January 2018. Wastewater samples were collected at the inlet of the WWTPs (behind the fine grid at the inlet and before the sedimentation tank) for seven consecutive days. Auto-samplers (Sigma-SD900, HACH Inc., Loveland, CO, USA) were used to collect time-proportional composite samples. The auto-samplers were programmed to collect one sample every 2 h (no less than 100 mL each time), and a composite sample was obtained by mixing the samples collected over a 24 h period. The same sampling procedure was used at all WWTPs. Samples were frozen at −20 °C at each WWTP immediately after composite sample collection. All samples were transported to the laboratory under frozen conditions and stored at −20 °C for less than one month until analysis. 

#### 2.1.2. Data of Responsible Officials

In the context of Chinese cities, each city is led by two key officials: the municipal party secretary and the mayor. The municipal party secretary, as the “top leader” of the prefecture-level city, is primarily responsible for party affairs, ensuring the effective implementation of the party’s guidelines and policies, and making decisions on major local social issues. The mayor, whose power is second only to that of the municipal party secretary, mainly handles the specific administrative affairs of the government. Besides the municipal party secretary and the mayor, the agricultural authorities and food safety authorities are also highly responsible for the residue of pesticides in wastewater from their jurisdictions. The agricultural authorities consist of officials from the Agriculture and Rural Affairs Bureau, who oversee agricultural policies and rural development initiatives. Meanwhile, the food safety in this study includes officials from the Food Administration, the Drug Administration, and the Health Commission, all of whom are responsible for ensuring food safety and maintaining public health standards.

This paper empirically investigates the effect of the political incentives of city officials on the provision of public urban food safety projects using wastewater data. Specifically, this study selected 50 officials from 32 major Chinese cities, ensuring that their jurisdictions precisely overlap with the 119 WWTPs where the wastewater samples were collected. The selected officials represent a diverse range of departments, including municipal leadership, agricultural authorities, and food safety authorities. This comprehensive selection of officials allows for a robust analysis of how knowledge backgrounds influence the provision of public urban food safety projects. This study defines an official’s background by their educational qualifications and work experience. Officials were considered to have an interdisciplinary knowledge background if they possessed a diploma, a bachelor’s degree, or higher in fields related to agriculture, pharmacology, medicine, public health, or a related disciplines. Officials were considered to have relevant professional experience if they had worked in sectors related to agriculture, healthcare, public health, or other relevant departments.

When we match the economic data of prefecture level cities with the official data, we follow the practice of the existing literature [16]. In this way, each prefecture level city matches a municipal party secretary and a mayor every year. We separate the entire sample of city officials into three subsamples—those younger than 55, those older than 55 but younger than 57, and those over 57 and approaching the age of 60. We take advantage of the ages of the officials and regard those under 55 years old as officials incentivized by their long-term development potential, those between 55 and 57 years old as promotion-incentivized officials, and those over 57 and approaching the age of 60 years old as low-incentive officials. In addition, we generate a dummy variable to distinguish whether the officials possess professional food education or a food-related working background.

The curriculum vitae information of the officials in the sampled cities was collected from the online public information of each prefecture-level city. In this study, data on the control variables including urban per capita GDP, urban population, the proportion of added value of secondary industry and tertiary industry in GDP, and the urban administrative area were referenced from the Statistical Yearbook of the corresponding cities, which can be downloaded or browsed online from the website of the China National Bureau of Statistics (https://data.stats.gov.cn/english/ (accessed on 12 May 2024)). Table 1 provides a summary of the descriptive statistics for the variables used in this study.

### 2.2. Lab Analysis

Sample pretreatment and analysis followed the standard method of the Determination of 331 Pesticides and Metabolites Residues: Liquid Chromatography-Tandem Mass Spectrometry Method (China National Standard, GB 23200.121-2021), with minor modifications according to the literature [25,26]. Briefly, the samples were passed through a glass filter before extraction, and deuterated internal standards (50 µL, 500 µg/L) were added to 50 mL filtered wastewater samples for quantification. Solid phase extraction (SPE) was used to extract the target analytes using OASIS HLB cartridges (3 cc/60 mg, Waters Corp., Milford, MA, USA).

The HLB cartridge was conditioned in sequence with MeOH (3 mL) and Milli-Q water (3 mL) at a rate of 5 mL/min. The spiked samples were loaded to the conditioned HLB cartridge under a vacuum at the same rate. The cartridges were dried under a nitrogen stream at a flow rate of 10 mL/min for 10 min and eluted with 5 mL of MeOH. The eluates were evaporated under a gentle nitrogen stream to below 0.5 mL and diluted to a constant volume of 0.5 mL with MeOH. A further filtration step was performed by a 0.2 µm centrifugal filter (modified nylon, VWR International, Radnor, PA, USA).

Chromatographic separation of the target compounds was conducted using an ultra-fast liquid chromatography (UFLC) system (20 AD-XR, Shimadzu, Kyoto, Japan) with a Phenomenex Gemini C18 column (100 mm × 2 mm, 3 µm) with an injection volume of 5 µL. The mobile phase was composed of 0.1% acetic acid in ultrapure water (A) and MeOH (B). Mass spectrometric analysis was conducted using an API-4000 triple quadrupole mass spectrometer (AB SCIEX, Framingham, MA, USA). The quantification of the MS system was operated in multiple reaction monitoring (MRM) mode. Two or three most abundant product ions of the protonated pseudo-molecular ion of each substance were chosen for analysis, which was done both in positive and negative ionization modes.

The analytical methods were subjected to strict quality assurance measures. The method detection limit (MDL) (S/N = 3) and method quantification limit (MQL) (S/N = 10) are summarized in Table S1 in the Supporting Information. The method was fully validated in raw wastewater.

### 2.3. Data Analysis

The daily mass loads of target pesticide residues (mg/day) at a specific WWTP were calculated according to the residue concentrations (ng/L) and the wastewater flow rate (m^3^/day) at the WWTPs. In order to compare results between different cities, the mass loads (mg/day) were normalized to the population served by each WWTP (mg/1000 inhabitants/day), using the following equation:

$$Load\;of\;pesticide\;residue=\frac{Concentration\times Influent\;flow}{Population}$$

where the load of pesticide residue (mg/1000 inhabitants/day) is the normalized index to characterize pesticide residues in the cities. Influent flows on each day of sampling were provided by the respective WWTPs. Populations served by WWTPs were either obtained from the WWTPs or based on the most recent census data of the service areas. Uncertainties involved in load estimation were discussed elsewhere [27].

To investigate the relationship between officials’ interdisciplinary backgrounds and urban pesticide residues, two dummy variables were constructed including whether (1) chief officials of agricultural authorities have food safety education or professional backgrounds, and whether (2) chief officials of food safety authorities have agricultural education or professional backgrounds. The least square method model with control variables was used to test the correlation between the load of pesticide residues in the urban wastewater samples and the interdisciplinary backgrounds of the officials. The regression equation is shown in formula (1):

(1) $$y_{psc}=\alpha_{psc}+\beta_{1}z_{c}+\gamma n_{c}+\varepsilon_{psc}$$

where *y_psc_* is the dependent variable, using the logarithm of the normalized load of pesticide residue (mg/1000 inhabitants/day) in urban wastewater. *Z_C_* is the main explanatory variable, using the dummy variable on “whether the chief officials of agricultural authorities have food safety education or professional backgrounds” or “whether the chief officials of food safety authorities have agricultural education or professional backgrounds”. When the officials have interdisciplinary backgrounds, *Z_C_* is 1. When the officials do not have interdisciplinary backgrounds, *Z_C_* is 0. *N_C_* includes the socio-economic variables that may affect the level of urban pesticide residues (urban per capita GDP, urban population, the proportion of the added value of secondary industry and tertiary industry in GDP, and the urban administrative area). *β*_1_ is the main estimation coefficient.

The least square method model with control variables was also used to test the correlation between the load of pesticide residues in wastewater in each city and the tenure cycle of the officials. The regression equation is shown in Formula (2):

(2) $$y_{psc}=\mu_{psc}+\beta_{2}x_{c}+\gamma v_{c}+e_{psc}$$

where *y_psc_* is the dependent variable, using the logarithm of the normalized load of pesticide residue (mg/1000 inhabitants/day) in urban wastewater. *X_C_* is the main explanatory variable, including three dummy variables: (1) the value of “Chief officials are under the age of 55” is 1, otherwise it is 0; (2) the value of “Chief officials are 55–57 years old” is 1, otherwise it is 0; and (3) the value of “Chief officials are over 57 and approaching the age of 60” is 1, otherwise it is 0. *V_C_* includes the socio-economic variables that may affect the level of urban pesticide residues (urban per capita GDP, urban population, the proportion of added value of secondary industry and tertiary industry in GDP, and the urban administrative area). *β*_2_ is the main estimation coefficient. 

## 3. Results and Discussion

### 3.1. Regional Distribution Characteristics of Pesticide Residues

**Figure 1:**
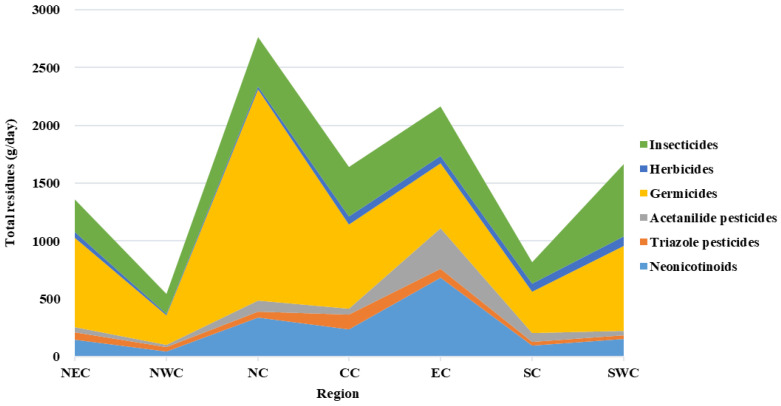
The residual pesticide components across the seven regions in China (NEC = Northeast China, NWC = Northwest China, NC = North China, CC = Central China, EC = East China, SC = South China, SWC = Southwest China).

From 2017 to 2018, samples were collected from 119 wastewater treatment plants in 32 cities in China. In the wastewater samples, 20 pesticides commonly used in China were detected, including neonicotine, triazole, amides, insecticides, fungicides, and herbicides. Data analysis showed, in terms of the spatial distribution across seven regions in China, the highest pesticide residues were found in North China, followed by East China. Neonicotinoid pesticides, fungicides, and insecticides are the three types of pesticides with the highest residues (Figure 1). In North China, the residual amount of fungicides is significantly higher than in other regions, accounting for 35% of all the seven regions. The amount of fungicides in North China accounts for 66% of the total residues of pesticides in this region, indicating that fungicides are the most important residual pesticides in cities in North China. In East China, the residual amount of neonicotinoid pesticides is significantly higher than in other regions (accounting for 40%). Fungicides and insecticides also have high residues in East China. In the total residual levels of the six types of pesticides in sewage from the seven regions, fungicides are the pesticide with the highest residual amount, accounting for 48% of the total pesticide residues. Next are insecticides (23%), followed by neonicotinoids (16%), indicating that these three types of pesticides are the most widely used varieties in China.

**Figure 2:**
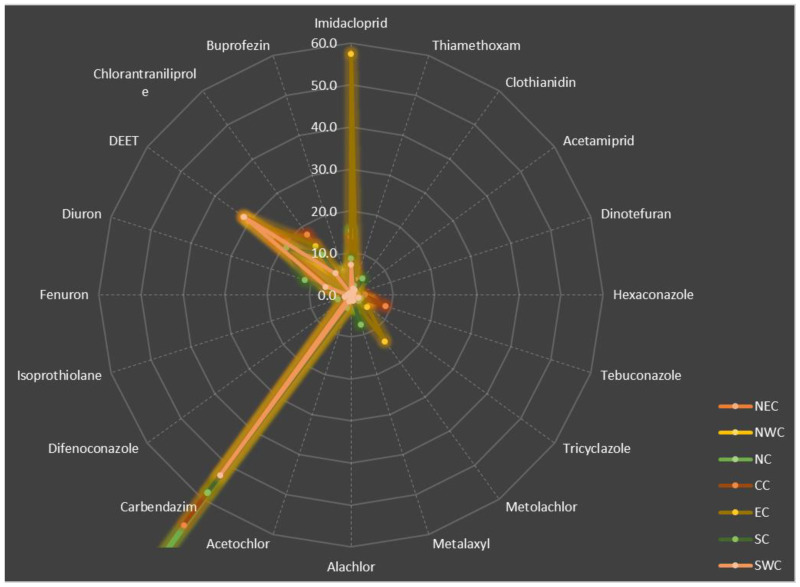
Pesticide residues per 1000 inhabitants across the seven regions in China.

Taking pesticide residues per 1000 inhabitants as an indicator for each region, the radar chart (Figure 2) showed the residual pesticide components across the seven regions. Carbendazim and diethyltoluamide (DEET) are the two types of pesticides with the highest residues in general. In addition to these two, each region has its own structural characteristics. For example, North China and East China have the most prominent pesticide residues per 1000 inhabitants. Among them, the residues of carbendazim in North China are significantly higher than those in other regions, mainly those from Shijiazhuang and Beijing with high residues of carbendazim. The residues per 1000 inhabitants of carbendazim in North China accounted for 72% of the total value of the residues per 1000 inhabitants in the region, making carbendazim the most significant residual pesticide in cities in North China. The residues of imidacloprid and metolachlor in East China were significantly higher than those in other regions, with imidacloprid mainly coming from Suzhou and metolachlor mainly coming from Shanghai. The sum of residues per 1000 inhabitants of imidacloprid and metolachlor accounted for 35% of the total in East China. In addition to carbendazim, DEET and imidacloprid, the residue of chlorantraniliprole is also prominent in the seven regions. Carbendazim is a fungicide, DEET and chlorantraniliprole are insecticides, and imidacloprid is a neonicotinoid pesticide. These outline the basic structure of pesticide use in vegetable and fruit production in China.

**Figure 3:**
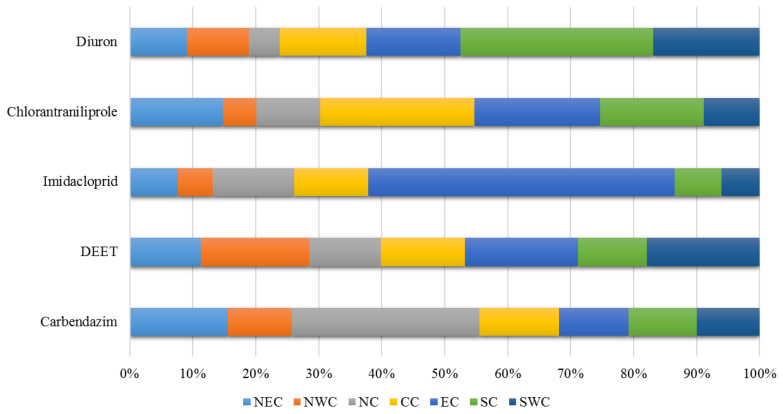
The regional distribution of the residues.

Regarding the 20 pesticides, the total amount of residues per 1000 inhabitants was 158.7 mg/1000 inh/day, of which carbendazim (76.1 mg/1000 inh/day) accounted for the largest proportion (48%). This was followed by DEET (25.1 mg/1000 inh/day, or 16%), imidacloprid (16.8 mg/1000 inh/day, or 11%), chlorantraniliprole (10.3 mg/1000 inh/day, or 6%), and diuron (5.4 mg/1000 inh/day, or 3%), cumulatively accounting for 85% of the total. The regional distribution of the total residues per 1000 inhabitants of these five pesticides is presented in Figure 3. DEET is evenly distributed in various regions, possibly because it is mainly used as a household mosquito repellent. Therefore, DEET in urban sewage mainly comes from cleaning and discharging unused products into the sewer. Imidacloprid is mainly distributed in East China, with Suzhou having the highest concentration of 495.04 mg/1000 inh/day, significantly higher than the national average. Carbendazim is mainly distributed in North China and Northeast China. The level of chlorantraniliprole is relatively high in Central and Eastern China. Diuron is mainly distributed in Southern China. The regional distribution of these pesticide residues reflects the structural characteristics of the types of pesticides exposed in daily life in different regions, which can provide valuable data guidance for targeted pesticide use control.

### 3.2. Relationship between Officials’ Interdisciplinary Background and Urban Pesticide Residues

According to the Food Safety Law, the responsibilities for the monitoring, testing, and risk assessment of the pesticides residues of food, from production to consumption, are on the shoulders of governmental agencies for agriculture, public health, and food safety risk management. Effective communication and information sharing between these organizations are crucial for the control of pesticides flows. In practice, the trans-agency knowledge, experiences, and attitudes of the officials in charge in these organizations may affect the effectiveness of their performances in pesticide control.

In this study, the curriculum vitae information of officials from agricultural and rural supervision departments and food supervision departments in the sampled cities were collected from online public information, and dummy variables were constructed including (1) whether the chief officials of agricultural and rural supervision departments have professional food education or food-related working backgrounds, and (2) whether the chief officials of food safety supervision departments have agricultural education or working backgrounds. A least squares model with control variables was used to test the correlation between the pesticide residue levels in the wastewater and the officers’ interdisciplinary cross-curricular background in the same city. Variables that may affect the content of pesticide residues in wastewater were set as control variables, including the urban administrative area, urban per capita GDP, urban population, and the proportion of added value of secondary industry and tertiary industry in GDP. The following hypothesis is put forward: the interdisciplinary knowledge background or professional experience of urban government officials plays an important role in the effect of urban pesticide control.

The results of regression analysis using dummy variables and control variables are summarized in Table 2. Dummy variable 1 (chief officials of agricultural management authorities with food safety education or professional backgrounds) has a significant negative correlation with the pesticide residues in wastewater. Similarly, the officials of food safety supervision departments with agricultural education or professional backgrounds (dummy variable 2) also have a significant negative correlation with the pesticide residues in wastewater. This shows that the interdisciplinary background of officials has a significant negative relationship with urban pesticide residues, which verifies our hypothesis; that is, the interdisciplinary knowledge background or professional experience of officials in related authorities plays an important role in the supervision and control of the effect of urban pesticide residues.

### 3.3. Officials’ Tenure Cycle and Urban Pesticide Residues

The theory of officials’ political career cycles is an interesting research field in political economics from the perspective of China. This theory holds that when officials are below the upper limit of the age range for career promotion, they are inclined to pay more attention to governance in non-economic fields, such as food safety governance. Generally, in China, the upper age range for promotion for the local chief administrative officials is 55–57. Within this age range, the chief administrative officials are most likely to be promoted due to the good performance of regional economic development.

In this section, the following hypothesis is put forward: when the chief administrative officials are 55–57 years old, they are most likely to focus on economic growth and pay less attention to the governance of social problems, such as food safety and pesticide residual control; young and middle-aged officials under the age of 55 have a relatively long political career and are more patient in dealing with social problems other than economic development; for officials over 57 and approaching the age of 60 who are about to retire, their motivation to develop the economy or solve social problems for promotion is relatively weak.

Similarly, the least square method model of control variables is used to test the correlation between the pesticide residue content in urban wastewater and the political career cycle of officials. Variables including the urban per capita GDP, urban population, and the proportion of added value of secondary industry and tertiary industry in GDP are set as control variables.

The results are summarized in Table 3. There is a significant negative correlation between the age of administrative officials being below 55 and the level of pesticide residues in wastewater, indicating that young and middle-aged officials are more patient in dealing with social problems rather than economic development. On the contrary, there is a significant positive correlation between the chief administrative officials being aged 55–57 and the level of pesticide residues in wastewater, indicating that the officials in this age group pay less attention to areas where the effectiveness of work is difficult to be observed. As expected, there is no significant correlation between officials over 57 and approaching the age of 60 and the levels of pesticide residues, which also verifies our previous conjecture: officials who are about to retire have relatively weak motivation to solve social problems.

It must be stressed that the potential of WBE in urban food safety and risk governance is not tapped yet. This study attempted to draw the attention of decision makers and other stakeholders to add value to WBE in future. 

## 4. Conclusions

Compared with over-ground infrastructure, underground infrastructure is often neglected by city governments all over the world. For example, drainage systems, which are less visible than roads and bridges, would have been probably underinvested in in both developed and developing countries. How do we encourage political officials to pay more attention to publicly important but over-ground, invisible provisions? This paper empirically investigates the effect of the political incentives of city officials on the provision of public urban food safety projects using wastewater data.

This study is closely related to the growing literature investigating the effects of local officials’ characteristics, especially education backgrounds, tenure, and age, on governance performance. We add to this literature by uncovering the impacts of the city officials’ professions, term, and age on the allocation of fiscal resources to invisible projects, especially to food safety.

In current institutional contexts of Chinese cities, it is important to place accountability on local officials that spans beyond their terms and draws their attention more to long-run, public-oriented development. The Chinese central government should redesign performance evaluation systems to rebalance the incentives of local officials across economic and social areas of responsibility. In the case of produce safety governance, WBE is an unsung hero who reveals the behaviors of pesticide residues and provides evidence for evaluating the performances of responsible managers.

The study employs pesticide residues in wastewater as a broad proxy for human exposure to pesticides, but this approach has notable limitations. Specifically, pesticide residues in wastewater can originate from diverse sources, including industrial discharges, agricultural runoff, and improper disposal, which complicates isolating the impact of each source on the observed residue levels. To address these limitations, future research should incorporate source apportionment techniques such as chemical fingerprinting and stable isotope analysis to distinguish between industrial, agricultural, and domestic origins of pesticide residues. Additionally, spatial analysis using GIS and buffer zone analysis can help identify correlations between residue concentrations and proximities to potential sources.

## Figures and Tables

**Table 1:** Descriptive statistics for the variables.

Variables	Obs.	Mean	Std. Dev.	Min	Max
Chief officials are under the age of 55	2618	0.4370	0.4961	0	1
Chief officials are 55–57 years old	2618	0.2017	0.4013	0	1
Chief officials are over the age of 57	2618	0.3613	0.4805	0	1
Population	2618	6.3866	0.6535	5.0814	8.1292
Proportion of added value of secondary industry in GDP	2618	0.3913	0.0973	0.1857	0.5298
Proportion of added value of tertiary industry in GDP Land area	2618	0.5737	0.1068	0.4285	0.8023
Per capita GDP	2618	11.3862	0.2937	10.8728	12.0282
Logarithm of the land area of administrative regions	2618	9.1892	0.8061	7.4378	11.3194
Logarithm of the number of hospital beds per capita	2618	4.2942	0.2176	3.8297	4.6027
Logarithm of the number of secondary vocational education teachers per capita	2618	2.0683	0.3341	1.5361	2.9066
Logarithm of the number of ordinary secondary school teachers per capita	2618	3.7807	0.1757	3.5144	4.3779

**Table 2:** Relationship between officials’ interdisciplinary backgrounds and urban pesticide residues.

Variables	Pesticide Residues in Wastewater.	
	Correlation Coefficient	Probability
Dummy variable 1		
Chief officials of agricultural authorities with food education or professional backgrounds	−0.138	0.045
Control variables		
Per capita GDP	0.414	0.094
Population	0.450	0.046
Proportion of added value of secondary industry in GDP	−0.055	0.011
Proportion of added value of tertiary industry in GDP	−0.056	0.010
Land area	−0.305	0.041
Dummy variable 2		
Chief officials of food authorities with agricultural education or professional backgrounds	−0.417	0.064
Control variables		
Per capita GDP	0.062	0.108
Population	0.531	0.047
Proportion of added value of secondary industry in GDP	−0.021	0.012
Proportion of added value of tertiary industry in GDP	−0.027	0.011
Land area	−0.355	0.041

**Table 3:** Relationship between officials’ tenure cycle and urban pesticide residues.

Variables	Pesticide Residues in Wastewater.	
	Correlation Coefficient	Probability
Dummy variable 1		
Chief officials are under the age of 55	−2.807	1.012
Control variables		
Per capita GDP	5.373	2.387
Population	1.689	1.127
Proportion of added value of secondary industry in GDP	0.263	0.269
Proportion of added value of tertiary industry in GDP	0.138	0.249
Land area	−0.046	1.039
Dummy variable 2		
Chief officials are 55–57 years old	5.769	1.302
Control variables		
Per capita GDP	2.424	2.529
Population	2.056	1.128
Proportion of added value of secondary industry in GDP	0.873	0.301
Proportion of added value of tertiary industry in GDP	0.741	0.280
Land area	1.095	0.995
Dummy variable 3		
Chief officials are over 57 and approaching the age of 60	−0.789	1.105
Control variables		
Per capita GDP	7.536	2.252
Population	1.216	1.117
Proportion of added value of secondary industry in GDP	0.358	0.295
Proportion of added value of tertiary industry in GDP	0.251	0.277
Land area	1.062	1.068

## Data Availability

The raw data supporting the conclusions of this article will be made available by the authors on request.

## Supplementary Materials

The following supporting information can be downloaded at: https://www.mdpi.com/article/10.3390/toxics12070504/s1, [28], Table S1: MDLs and MQLs of the target compounds; Table S2: Load of pesticide residues in each region (mg /1000 inhabitants/day); Table S3: Concentrations of pesticide residues (ng/L).
