# Supplementary material for: Towards Evidence-Based Food Safety Governance with Wastewater-Based Epidemiology (WBE) Technology in China
Source: Toxics. 2024 Jul 12;12(7):504. doi: 10.3390/toxics12070504 (PMC11281293; doi:10.3390/toxics12070504)
Supplement: Supplementary file 1 [file toxics-12-00504-s001.zip › toxics-3072568-supplementary.pdf]

toxics MDPI

Supplementary materials

S1. Lab analysis

In this study, 22 kinds of pesticides commonly used in China were tested, including *Neonicotinoids*, *Triazole pesticides*, Acetanilide pesticides, Germicides, herbicides and Insecticides. Sample pretreatment and lab analysis followed the standard method of the China National Standard: Determination of 331 Pesticides and Metabolites Residues Liquid Chromatography-Tandem Mass Spectrometry Method (GB 23200.121-2021). This method can be browsed online or downloaded from the website https://www.chinesestandard.net/PDF/English.aspx/GB23200.121-2021.

The GB 23200.121-2021 standard provides a comprehensive method for analyzing pesticide residues. Here is a summary of the key components of the English version of the detection method:

Scope of the Standard: The standard is applicable to the determination of 331 types of pesticides and their metabolites.

List of Pesticides and Metabolites: It includes a detailed list of the 331 pesticides and their metabolites, with their English and Chinese names, and specifies the quantification limits for each compound.

Sample Preparation: The standard mentions the QuEChERS (Quick, Easy, Cheap, Effective, Rugged, and Safe) method for sample preparation, which is a rapid extraction technique suitable for LC-MS/MS analysis.

Chromatographic Conditions: The standard details the conditions for liquid chromatography, including the type of column, mobile phase, flow rate, and gradient elution program.

Mass Spectrometry Conditions: It specifies the conditions for tandem mass spectrometry, such as the ionization mode (ESI+ or ESI-), capillary voltage, desolvation temperature, and collision energy for each pesticide and metabolite.

Multiple Reaction Monitoring (MRM): The standard provides MRM transitions for each pesticide and metabolite, which include the quantitative and qualitative ions used for detection and quantification.

Data Analysis: It outlines the method for data processing and analysis, including the integration of peak areas, calibration curve construction, and the calculation of recovery rates.

Quality Control: The standard includes procedures for quality control, such as the use of spiked samples, matrix-matched calibration, and the assessment of method performance with recovery rates and precision.

Method Validation: It provides criteria for method validation, including sensitivity, accuracy, precision, and specificity.

The method detection limit (MDL) and method quantification limit are summarized in Table S1. Among the 22 pesticides, 20 were detected in wastewater samples, and thiacloprid and nitenpyram were not detected in all samples.

Table S1. MDLs and MQLs of the target compounds.

No.	Pesticide	CAS	Category	MDL (ng/L)	MQL (ng/L)
1	Imidacloprid	138261-41-3	*Neonicotinoids*	0.04	0.15
2	Thiamethoxam	153719-23-4	*Neonicotinoids*	0.07	0.22
3	Clothianidin	210880-92-5	*Neonicotinoids*	0.05	0.17
4	Acetamiprid	135410-20-7	*Neonicotinoids*	0.04	0.12
5	Thiacloprid	111988-57-1	*Neonicotinoids*	0.01	0.02
6	Dinotefuran	165252-70-0	*Neonicotinoids*	0.16	0.52
7	Nitenpyram	133112-33-7	*Neonicotinoids*	0.05	0.18

8	Hexaconazole	70124-51-9	*Triazole pesticide*	0.07	0.22
9	Tebuconazole	107534-96-3	*Triazole pesticide*	0.10	0.34
10	Tricyclazole	94361-02-7	*Triazole pesticide*	0.03	0.10
11	Metolachlor	51218-45-2	Acetanilide pesticide	0.19	0.65
12	Metalaxyl	52918-63-5	Acetanilide pesticide	0.01	0.04
13	Alachlor	15972-60-8	Acetanilide pesticide	0.25	0.82
14	Acetochlor	34256-82-1	Acetanilide pesticide	0.17	0.55
15	Carbendazim	1563-66-2	Germicide	0.07	0.25
16	Difenoconazole	119446-68-3	Germicide	0.05	0.15
17	Isoprothiolane	27554-26-3	Germicide	0.02	0.06
18	Fenuron	330-54-1	Herbicide	0.03	0.11
19	Diuron	330-55-2	Herbicide	0.62	2.08
20	Diethyltoluamide (DEET)	134-62-3	Insecticide	0.01	0.02
21	Chlorantraniliprole	164285-71-8	Insecticide	1.08	3.60
22	Buprofezin	68392-35-8	Insecticide	0.003	0.01

S2. Data set

S2.1 Load of pesticide residues

Wastewater samples were collected from 32 cities. According to the division of the seven regions in China, the loads of pesticide residues (mg /1000 inhabitants/day) in each region are calculated by using the mean value of the cities in the region. Because the population-normalized load of pesticide residues (mg /1000 inhabitants/day) is not affected by the population difference, it is suitable for comparison between cities and regions. The load of pesticide residues in each region is summarized in Table S2.

Table S2. Load of pesticide residues in each region (mg /1000 inhabitants/day).

Category	Pesticide	NEC	NWC	NC	CC	EC	SC	SWC
Neonicotinoids	Imidacloprid	9.0	6.5	15.3	13.8	57.4	8.7	7.2
Neonicotinoids	Thiamethoxam	3.6	1.6	2.6	4.4	3.7	2.4	1.6
Neonicotinoids	Clothianidin	2.2	1.7	2.4	2.4	2.5	4.7	1.4
Neonicotinoids	Acetamiprid	0.8	0.1	0.4	1.9	0.3	0.9	0.2
Neonicotinoids	Dinotefuran	1.2	0.2	0.2	0.3	1.3	0.2	0.3
	Subtotal	16.8	10.2	20.9	22.8	65.3	16.9	10.8
Triazole pesticide	Hexaconazole	0.5	0.5	0.6	3.3	1.6	0.4	0.5
Triazole pesticide	Tebuconazole	5.3	4.5	2.3	8.6	2.5	2.2	1.9
Triazole pesticide	Tricyclazole	0.3	0.0	0.0	0.7	4.8	2.3	0.5
	Subtotal	6.1	5.0	2.9	12.5	8.9	4.9	2.9
Acetanilide pesticide	Metolachlor	0.4	0.0	0.0	0.2	13.7	0.3	0.1
Acetanilide pesticide	Metalaxyl	0.9	3.3	2.8	1.4	1.0	7.4	1.5
Acetanilide pesticide	Alachlor	0.2	0.0	0.0	0.0	0.0	0.0	0.0
Acetanilide pesticide	Acetochlor	3.7	2.1	2.5	3.2	2.5	3.2	1.6
	Subtotal	5.2	5.4	5.3	4.7	17.1	10.9	3.2
Germicide	Carbendazim	82.8	54.1	158.8	67.7	58.3	58.0	53.1
Germicide	Difenoconazole	0.1	1.4	0.2	1.4	0.2	0.2	0.3
Germicide	Isoprothiolane	3.1	0.8	0.5	1.2	1.9	3.3	1.6
	Subtotal	86.1	56.3	159.4	70.3	60.5	61.6	55.0
Herbicide	Fenuron	2.5	0.7	0.6	1.0	1.2	1.5	0.9
Herbicide	Diuron	3.4	3.7	1.8	5.1	5.6	11.5	6.3

	Subtotal	5.9	4.4	2.4	6.2	6.8	13.0	7.2
Insecticide	Diethyltoluamide (DEET)	19.8	30.2	20.0	23.4	31.7	19.1	31.4
Insecticide	Chlorantraniliprole	10.7	3.9	7.3	17.7	14.4	12.0	6.4
Insecticide	Buprofezin	0.9	5.9	0.9	0.3	0.3	0.5	0.4
	Subtotal	31.4	39.9	28.2	41.4	46.5	31.6	38.2
Total		151.6	121.2	219.1	157.9	205.0	138.9	117.3

S2.2 Concentration of pesticide residues

The pesticide residue concentrations in the seven regions are summarized in Table S3.

Table S3. Concentrations of pesticide residues (ng/L).

Category	Pesticide	NEC	NWC	NC	CC	EC	SC	SWC
Neonicotinoids	Imidacloprid	21.1	24.3	44.2	31.2	27.5	25.1	21.7
Neonicotinoids	Thiamethoxam	7.8	5.1	8.8	9.6	11.0	7.0	4.5
Neonicotinoids	Clothianidin	4.8	6.6	8.3	5.4	8.0	14.0	4.8
Neonicotinoids	Acetamiprid	2.3	0.5	1.3	6.2	1.0	2.4	0.6
Neonicotinoids	Dinotefuran	2.7	0.9	0.4	0.8	5.0	0.5	0.5
	Subtotal	38.6	37.4	63.1	53.1	52.6	49.0	32.2
Triazole pesticide	Hexaconazole	1.5	1.5	1.9	8.0	4.5	1.2	1.4
Triazole pesticide	Tebuconazole	13.4	12.8	5.3	22.3	7.9	6.6	6.3
Triazole pesticide	Tricyclazole	0.9	0.1	0.0	1.2	12.8	7.0	2.0
	Subtotal	15.8	14.3	7.2	31.5	25.2	14.8	9.6
Acetanilide pesticide	Metolachlor	1.1	0.0	0.0	0.5	2.3	1.0	0.5
Acetanilide pesticide	Metalaxyl	2.1	12.9	6.9	3.2	3.1	23.0	4.6
Acetanilide pesticide	Alachlor	0.4	0.0	0.0	0.0	0.0	0.0	0.0
Acetanilide pesticide	Acetochlor	8.8	7.5	8.4	7.3	7.9	9.9	5.0
	Subtotal	12.3	20.5	15.3	11.0	13.3	33.8	10.2
Germicide	Carbendazim	196.3	185.9	528.8	161.8	191.1	170.1	153.1
Germicide	Difenoconazole	0.3	3.9	0.6	3.8	0.7	0.6	1.0
Germicide	Isoprothiolane	9.0	2.8	1.9	2.6	5.8	10.3	4.9
	Subtotal	205.7	192.5	531.3	168.2	197.5	181.1	159.0
Herbicide	Fenuron	5.7	2.4	2.1	2.3	3.4	4.4	2.9
Herbicide	Diuron	9.5	14.0	6.7	10.8	17.0	33.9	17.1
	Subtotal	15.2	16.4	8.8	13.0	20.4	38.2	20.1
Insecticide	Diethyltoluamide (DEET)	44.3	107.6	67.2	55.8	100.6	56.1	95.9
Insecticide	Chlorantraniliprole	23.9	14.0	24.1	46.3	43.6	36.3	19.4
Insecticide	Buprofezin	2.0	15.7	2.3	0.7	1.1	1.6	1.1
	Subtotal	70.2	137.3	93.6	102.8	145.2	93.9	116.4
Total		357.8	418.3	719.2	379.7	454.3	410.8	347.5
